# Opening a Window into Accessory Pathway Mapping in Children with Wolff–Parkinson–White Syndrome?

**DOI:** 10.19102/icrm.2022.13114

**Published:** 2022-11-15

**Authors:** William Regan, Kate Harris, Eric Rosenthal

**Affiliations:** ^1^Evelina London Children’s Hospital, Guys and St Thomas’ NHS Foundation Trust, London, UK

**Keywords:** Ablation, accessory pathway, open-window mapping, pediatric, Wolff–Parkinson–White

## Abstract

We discuss the use of an open-window mapping technique to define the accessory pathway location in a child presenting with symptomatic Wolff–Parkinson–White (WPW) syndrome. This technique may have important applications for children with WPW syndrome and can be carried out using conventional mapping catheters.

A 12-year-old girl with a history of palpitations was found to have a narrow-complex, short-RP tachycardia on ambulatory monitoring and a normal resting 12-lead electrocardiogram (ECG) with no ventricular pre-excitation. She was intolerant of β-blocker therapy and, in consultation with her family, elected for an electrophysiology study and ablation.

The first procedure was carried out under general anesthesia using a fluoroscopy-free approach and the CARTO™ 3-dimensional electroanatomic mapping system (Biosense Webster, Diamond Bar, CA, USA). The baseline ECG showed ventricular pre-excitation (not previously seen) with a short H–V interval of 10 ms. Isoprenaline induced atrioventricular (AV) re-entry tachycardia (AVRT) in keeping with her clinical arrhythmia via a right lateral accessory pathway (AP). Radiofrequency (RF) ablation was performed using a solid-tipped Navistar™ catheter (Biosense Webster) at a site of early atrial signals during ventricular pacing **(Figure 1)**, which resulted in a transient loss of AP conduction. Following the recurrence of ventriculoatrial (VA) conduction across the AP, further RF ablation was performed using an irrigated SmartTouch™ catheter (Biosense Webster). However, neither tachycardia elimination nor retrograde AP conduction was achievable, despite a long procedure with >4 min of RF ablation. The procedure was terminated, and the child was restarted on anti-arrhythmic therapy.

Later, due to a recurrence of symptoms, she was brought back for a repeat procedure, which was also carried out under general anesthesia using a fluoroscopy-free approach and the CARTO™ 3-dimensional electroanatomic mapping system. VA conduction via a right lateral AP was demonstrated, and AVRT was easily induced. The tricuspid valve annulus was mapped with the ablation catheter using the “open-window” technique described by Schricker et al.^1^ The resulting map showed a relatively broad area of breakthrough to the atrium during ventricular pacing along a 12-mm length of the tricuspid valve annulus **(Figure 2)**, with similar conventional electrogram (EGM) markers of “early” atrial activation and fused VA signals found along the same length of the tricuspid annulus. RF ablation using an irrigated SmartTouch™ catheter at 40 W was carried out from the center of the breakthrough, with consolidation lesions on either side to form a short, linear lesion along the area of breakthrough at the tricuspid valve annulus (contrast this with the extensive RF lesion set on the first procedure, **Figure 1**). RF ablation resulted in a VA block and no inducible tachycardia. The child remained symptom-free during follow-up at 5 months with no ventricular pre-excitation on a 24-h Holter monitor.

We herein outline the use of open-window mapping (OWM) for a recurrence of a right-lateral AP in a child with WPW syndrome and documented AVRT. Ablating APs on the right AV groove can be complicated by stability on the AV ring and interpretation of the EGMs. Ineffective test lesions may be due to poor contact with or imprecise location on the AV ring. The OWM technique automatically annotates local activation, collecting points on either side of the AV ring irrespective of whether they are positioned in the atria or ventricles.^1^ The CARTO™ 3 Wavefront Annotation algorithm uses the sharpest unipolar signal (maximum dV/dt) timed with the local bipolar signal on the mapping catheter. An extended early-meets-late line demarcates the AV annulus, and the window of interest can be adjusted to exclude signals originating from a single chamber (ie, excluding ventricle signals when mapping during ventricular pacing), which allows the visualization of the “breakthrough” across the AV ring at the site of the AP. This may have advantages over conventional methods of defining AP conduction that rely on manually annotating the timing of early atrial, ventricular, or AP signals, as manual EGM interpretation can be subjective and frequently “good” and “excellent” signals can be found over a wide area. In our patient, it particularly offered an additional way to map, as her previous ablation hampered EGM interpretation. The OWM technique can be seen as an addition to conventional methods, allowing fast mapping to visualize a breakthrough area on the AV groove, but still allowing the operator to target conventional signals within the area of interest. Early reports of OWM have typically used high-density mapping catheters for rapid collection of points^1,2^; however, in children, catheter selection is also influenced by patient size. We outline the use of the OWM technique using conventional mapping catheters with a higher concentration of points and therefore good spatial resolution in the area of interest. Additional applications of the technique to consider are mapping in both directions with the AP capable of both antegrade and retrograde conduction, helping to define APs with broad insertion points (as seen in Ebstein’s anomaly of the tricuspid valve) or those with an oblique course across the AV valve annulus.^3^ Further use is required to see whether it remains an additional tool to supplement conventional EGM interpretation and standard EA mapping or is superior.

## Figures and Tables

**Figure 1:**
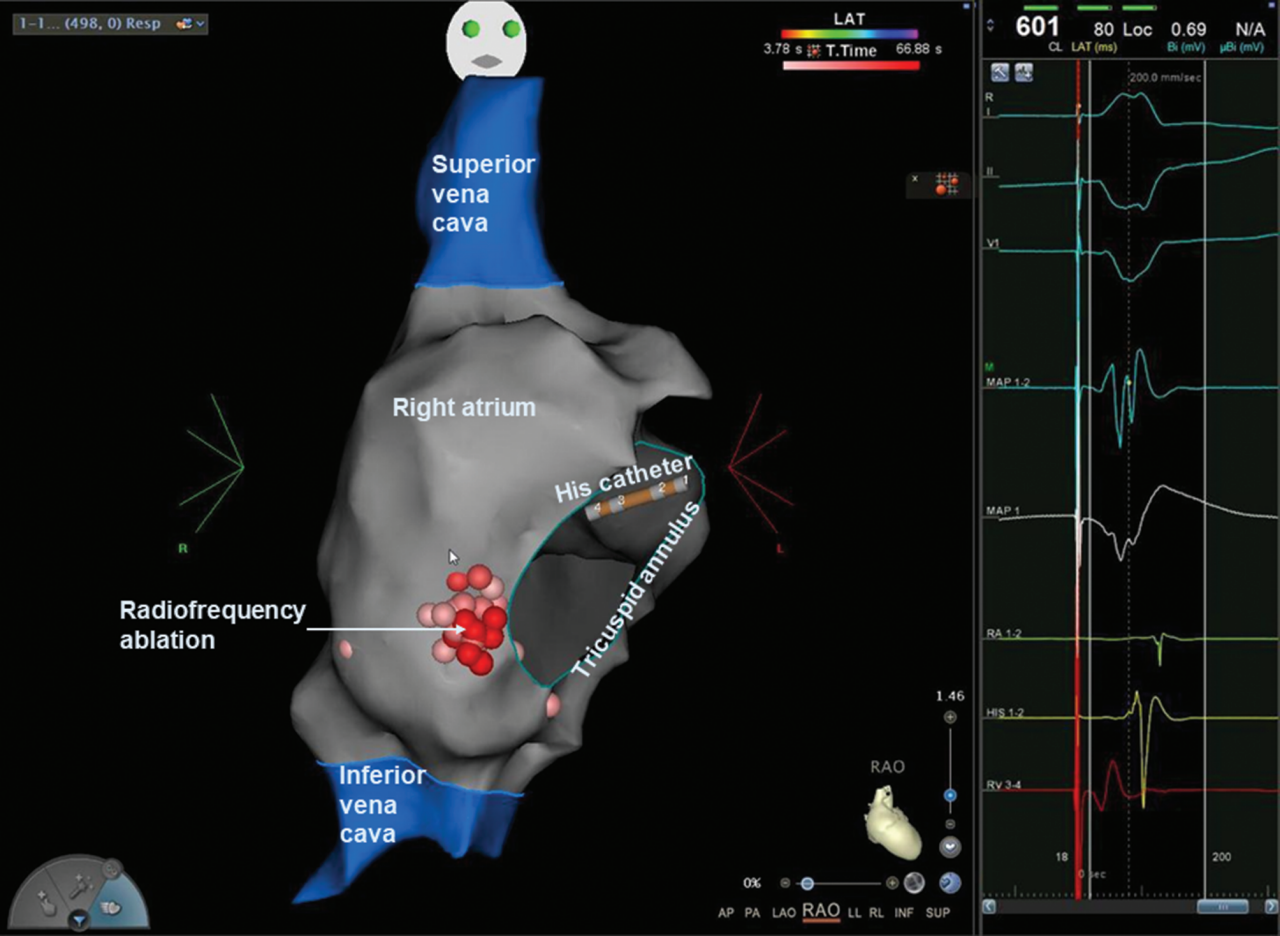
Right atrial geometry in the right anterior oblique projection highlighting the sites of radiofrequency ablation. **Right:** Intracardiac electrograms showing signals on the ablation catheter (bipolar signals on “MAP 1,2” and unipolar on “MAP 1”) at the site of radiofrequency ablation with early atrial and fused ventriculoatrial signals during ventricular pacing.

**Figure 2:**
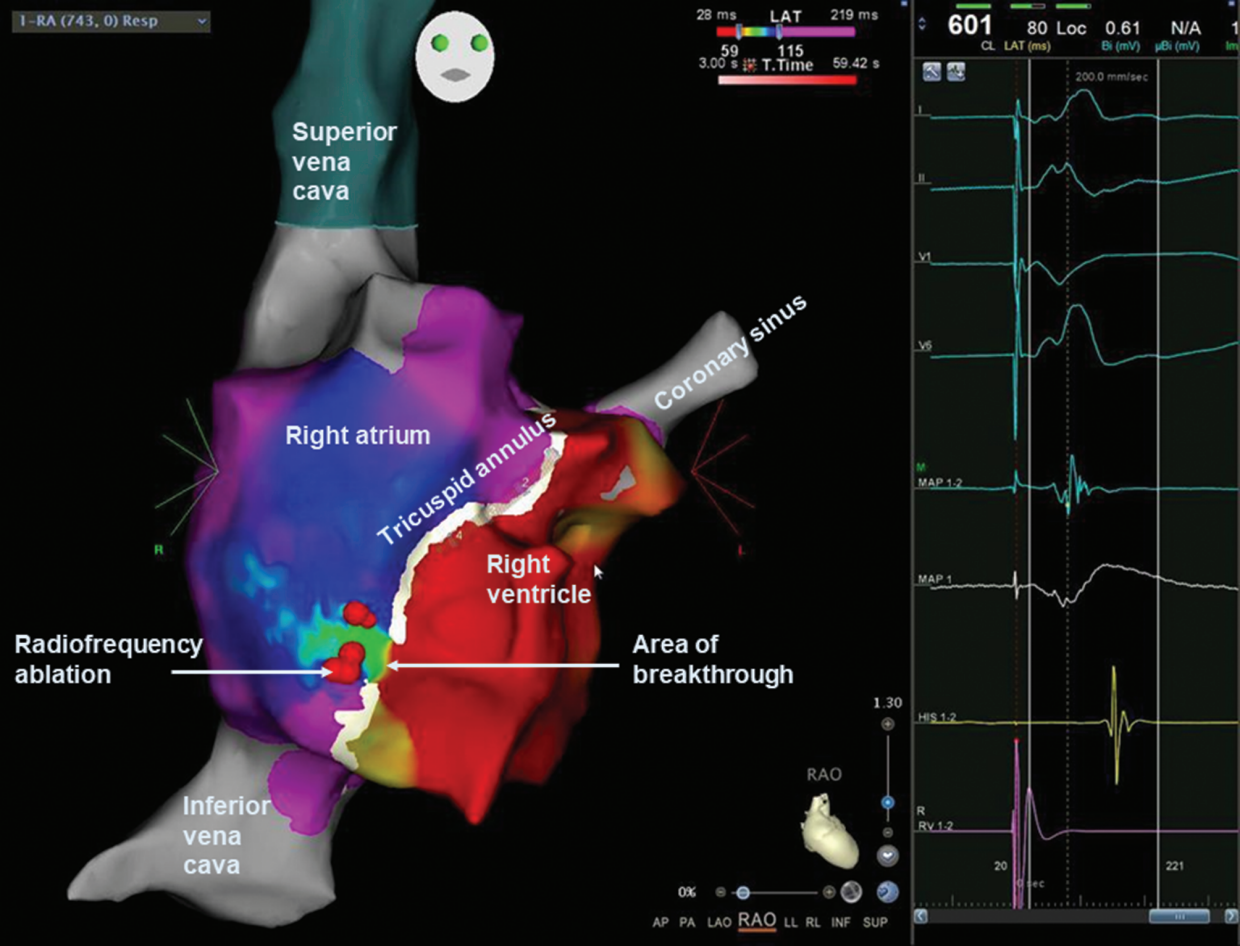
Right atrium in the right anterior oblique projection, with open-window mapping showing the breakthrough of atrial activation during ventricular pacing. **Right:** Intracardiac electrograms showing signals on the ablation catheter (bipolar signals on “MAP 1,2” and unipolar on “MAP 1”) at the site of radiofrequency ablation.
